# Report and Comparative Genomics of an NDM-5-Producing *Escherichia coli* in a Portuguese Hospital: Complex Class 1 Integrons as Important Players in *bla*_NDM_ Spread

**DOI:** 10.3390/microorganisms10112243

**Published:** 2022-11-12

**Authors:** Rafael D. S. Tavares, Marta Tacão, Elmano Ramalheira, Sónia Ferreira, Isabel Henriques

**Affiliations:** 1 Centre for Functional Ecology (CFE), Department of Life Sciences, University of Coimbra, Calçada Martim de Freitas, 3000-456 Coimbra, Portugal; 2 Centre for Environmental and Marine Studies (CESAM) and Department of Biology, University of Aveiro, Campus Universitário Santiago, 3810-193 Aveiro, Portugal; 3 Serviço de Patologia Clínica, Centro Hospitalar do Baixo Vouga-EPE, Avenida Artur Ravara, 3810-501 Aveiro, Portugal; 4 Department of Medical Sciences, University of Aveiro, Campus Universitário Santiago, 3810-193 Aveiro, Portugal; 5 Instituto de Educação e Cidadania, Largo da Igreja, Mamarrosa, 3770-033 Aveiro, Portugal

**Keywords:** integrons, carbapenemases, New Delhi metallo-beta-lactamase, clinical strain, *E. coli* ST156, FIB-FII plasmid

## Abstract

Background: New Delhi metallo-beta-lactamase (NDM) has been spreading across the globe, but the causes of its success are poorly understood. We characterized a *bla*_NDM-5_-positive *Escherichia coli* strain from a Portuguese hospital and conducted comparative genomic analyses to understand the role of clonal background and horizontal gene transfer in *bla*_NDM-5_ dissemination. Methods: After *bla*_NDM_ PCR screening and genome sequencing, Ec355340 was subjected to mating, transformation, and plasmid curing assays and MICs determination for several antibiotics. Comparison with data compiled from public databases was performed. Results: *bla*_NDM-5_ was in a complex integron co-located in a FIB-FII plasmid (pEc355340_NDM-5). The mating assays were unsuccessful, but plasmid transformation into a susceptible host led to resistance to all beta-lactams and to sulfamethoxazole-trimethoprim. The profile of virulence genes (n = 73) was compatible with extraintestinal pathogenesis. An analysis of genomes from public databases suggested that *bla*_NDM-5_ has rarely been associated with ST156 strains (such as Ec355340), while is has frequently been found on strains of the ST10 clonal complex. However, ST156 may play a role in the co-spreading of *bla*_NDM_ and *mcr* genes. Regardless, comparative genomics confirmed the presence of *bla*_NDM_ in similar complex integrons in plasmids (48/100 plasmids most similar to pEc355340_NDM-5) and ST156 genomes (20/41 *bla*_NDM_-positive genomes). Conclusions: *bla*_NDM-5_ and other *bla*_NDM_ variants were more frequently associated to complex integrons than previously reported and, therefore, these platforms may be important drivers in their dissemination. The identification of *bla*_NDM-5_ for the first time in Portugal could be a game-changer in the current Portuguese antibiotic resistance scenario, as this gene encodes a higher-level resistance phenotype, and its spread may be facilitated due to the association with complex integrons.

## 1. Introduction

Antibiotic resistance constitutes one of the most serious threats to modern medicine since it compromises the treatment of bacterial infections and hinders the prophylactic regimens necessary for several medical procedures [1]. Even resistance to last-resort antibiotics such as carbapenems has emerged, mostly through the dissemination of carbapenemase genes [2]. Carbapenem-resistant *Enterobacteriaceae* (CRE) have been recognized as a priority target group for the development of novel therapeutic agents [3], with higher mortality rates (two- to three-fold higher) having been reported for infections caused by CRE comparative to infections provoked by carbapenem-susceptible *Enterobacteriaceae* [4].

New Delhi metallo-beta-lactamase (NDM) was first reported in an isolate from a Swedish patient with previous hospitalization in India [5]. Initially, most cases reported in Europe were imported from the Indian subcontinent [6], though currently this enzyme has spread worldwide and is endemic to southeast Asia [7,8]. Strains harbouring *bla*_NDM_ are resistant to most beta-lactams (which NDM enzymes can hydrolyse) and to other classes of antibiotics due to the concomitant carriage in the same plasmids of several antibiotic resistance genes (ARGs) [6]. To date, 42 NDM variants have been described (Beta-Lactamase Database, http://bldb.eu/ accessed on 1 January 2022), and the most common *bla*_NDM_ carriers are *Klebsiella pneumoniae* and *Escherichia coli* [8].

NDM has been reported to be the third most prevalent carbapenemase worldwide [9], and according to recent reviews, Europe is the continent with the second highest number of reports for NDM [10,11]. Several outbreaks of NDM-producing bacteria in European countries have been reported [8], yet in Portugal *bla*_NDM_ has been described only sporadically [12,13,14,15,16]. In most reports in Portuguese settings, *bla*_NDM-1_ has been found to be associated with IncC plasmids either from clinical *Morganella morganii*, *Proteus mirabilis* and *Providencia stuartii* strains [12,13], or from an environmental *Enterobacter roggenkampii* isolate [14]. However, a multi-replicon IncR-FIA plasmid carrying *bla*_NDM-1_ was also described in a *K. pneumoniae* outbreak in a Portuguese hospital [16].

NDM-5 was first described in an *E. coli* strain from a patient in the UK [17] and it is considered an emerging carbapenemase, particularly in *E. coli,* where it appears to be the predominant NDM variant [8,18]. NDM-5 has two substitutions in its amino acid sequence, Val88→Leu and Met154→Leu, comparative to NDM-1, which leads to higher resistance levels to carbapenems (eight-fold higher for meropenem and at least four-fold for ertapenem and imipenem) [11]. The gene encoding NDM-5 is usually associated with IncX3 or IncF plasmids [8,18].

In this study, our aim was to characterize the first *bla*_NDM-5_-positive *E. coli* strain (Ec355340) identified in a Portuguese nosocomial environment. To add knowledge on the contribution of the clonal background and/or lateral gene transfer to NDM dissemination worldwide, the analysis was extended to other genomes retrieved from public databases.

## 2. Materials and Methods

### 2.1. Bacterial Isolation, Antibiotic Susceptibility Testing and bla_NDM_ Detection

As part of the routine surveillance program of a hospital in the north of Portugal, a carbapenem-resistant strain was retrieved from a urine sample of a patient admitted in 2019 with a urinary tract infection. The strain was identified by VITEK^®^ 2 (bioMérieux, Craponne, France) and named Ec355340. Minimum inhibitory concentrations (MICs) for ampicillin, amoxicillin/clavulanic acid, piperacillin/tazobactam, cefuroxime, cefuroxime axetil, cefotaxime, ceftazidime, cefepime, ertapenem, meropenem, amikacin, gentamicin, ciprofloxacin, fosfomycin, nitrofurantoin, colistin and sulfamethoxazole-trimethoprim were determined by VITEK^®^ 2 (AST-N355 card, bioMérieux, Craponne, France). PCR detection of *bla*_NDM_ was performed as described elsewhere (Table S1).

### 2.2. Whole-Genome Sequencing and Analysis

Genomic DNA was obtained using the Wizard^®^ Genomic DNA Purification Kit (Promega, Madison, WI, USA) and sequenced with the Illumina HiSeq 2500 platform (Illumina, San Diego, CA, USA). The raw reads were processed following a previously reported pipeline [14].

Automatic annotation was performed by RAST (https://rast.nmpdr.org/rast.cgi). Specific genomic features (i.e., ARGs, virulence genes (VGs), plasmid replicons and multi-locus sequence typing (MLST)) were analysed using tools from the Center for Genomic Epidemiology (https://cge.cbs.dtu.dk/services/), The Comprehensive Antibiotic Resistance Database (https://card.mcmaster.ca/home) and the Virulence Factor Database (http://www.mgc.ac.cn/VFs/) as previously described [19].

### 2.3. Plasmid Analysis

The assembly of the *bla*_NDM_-carrying plasmid from multiple contigs was achieved by PCR amplification of the gap regions (Table S1) followed by Sanger sequencing (Eurofins Genomics, Ebersberg, Germany).

Conjugal transfer was attempted by mating assays in liquid media using rifampicin-resistant *E. coli* CV601 as the recipient, with a donor/recipient ratio of 1:1 [19]. Transconjugants were selected on LA agar (NZYTech, Lisboa, Portugal) supplemented with meropenem (1 µg/mL) and rifampicin (100 µg/mL, Sigma-Aldrich, St. Louis, MO, USA).

Plasmid DNA was extracted using the Qiagen Plasmid Mini Kit (Qiagen, Hilden, Braine-l’AlleudGermany) and electroporated into TOP10 Electrocomp™ *E. coli* cells following the manufacturer’s instructions (Invitrogen, Waltham, MA, USA).

Transconjugants and transformants were confirmed by PCR detection of *bla*_NDM_ and BOX-PCR fingerprinting (Table S1). Plasmid DNA was digested with *Bst*1107I and *Pst*I (Thermo Scientific, Waltham, MA, USA) as previously described [19]. MICs were determined as described above for the same set of antibiotics.

Plasmid curing was attained by 10-daily successive passages in non-selective LB medium (approximately 700 generations). An initial inoculum of 10^4^–10^5^ cells/mL was incubated in 12-well microplates (Orange Scientific, Belgium) at 37 °C in static conditions. Daily passages were performed by serial dilution of each culture to 10^4^–10^5^ cells/mL in fresh medium. After 10 passages, cultures were diluted and plated in MacConkey Agar (VWR, Radnor, PA, USA). Randomly selected colonies were screened for *bla*_NDM_, and negative colonies were further characterized by BOX-PCR (comparatively to the original strain) and by PCR screening of *intI1* and ARGs previously identified by genome sequencing (Table S1). To confirm if the *bla*_NDM_-containing plasmid was lost or simply rearranged, DNA of a *bla*_NDM_-negative strain was sent for whole-genome sequencing. MICs were determined for this strain.

### 2.4. Comparative Analyses of ST156 and bla_NDM-5_-Harbouring Genomes

*E. coli* ST156 genomes and corresponding metadata were retrieved from the PATRIC database (August 2020, https://www.patricbrc.org/ accessed on 1 January 2022). Each genome was analysed as described above in terms of ST, ARGs and VGs content. Phylogenetic relationships were established by single nucleotide polymorphism (SNP) analysis using REALPHY 1.12 with the default parameters (https://realphy.unibas.ch/). To perform hierarchical analysis based on presence/absence of ARGs, VGs and plasmid profiles, similarity matrixes were built using the Jaccard coefficient in Primer v.6 (Primer-E Ltd., Lutton, UK). The genetic context of *bla*_NDM_ genes was manually annotated. To assess the association between complex integrons and *bla*_NDM_, an *intI1* sequence (NC_022375.1) was used as the query in a BLAST search against a custom database composed of the ST156 genomes’ dataset. The structure and gene content of these integrons were manually inspected.

Additionally, *bla*_NDM-5_ positive genomes available in PATRIC were selected by accessing the “PATRIC Local Family” of the genome feature “NDM-5” (genomes containing other *bla*_NDM_ variants were excluded), and their metadata were extracted for analysis of their isolation source and sequence type (ST).

## 3. Results

### 3.1. Genotype and Phenotype of Ec355340

Strain Ec355340 was identified by VITEK^®^ 2 as an *E. coli* strain, displaying a multiresistance phenotype to all beta-lactams tested, ciprofloxacin and the combination sulfamethoxazole-trimethoprim (Table 1). It remained susceptible to aminoglycosides, colistin, fosfomycin and nitrofurantoin (Table 1). *bla*_NDM_ carriage was confirmed by PCR.

The isolate was characterized by whole-genome sequencing (quality metrics are presented in Table S2). *In silico* typing affiliated Ec355340 to *E. coli* ST156 (Warwick MLST scheme) and serotype O54:H28. PathogenFinder indicated this strain as a potential human pathogen with a probability of 93.4%.

ResFinder-based screening identified nine ARGs that conferred resistance to beta-lactams (*bla*_NDM-5_, *bla*_TEM_-like), fluoroquinolones (*qepA4*), macrolides (*mdfA*, *mphA*), aminoglycosides (*aadA2*), sulphonamides (*sul1*), tetracyclines (*tetB*) and trimethoprim (*dfrA12*). Mutations in *parC* (Ser80→Ile and Glu84→Gly) and *gyrA* (Ser83→Leu and Asp87→Asn), known to be responsible for resistance to fluroquinolones, were also identified. The combined use of VirulenceFinder and VFanalyzer led to the detection of 73 VGs, most of which were associated to bacterial adhesion (39.7% of the VGs detected, e.g., *ipfA* and *eaeH*), secretion systems (23.3%, i.e., genes associated to type VI secretion systems) and iron acquisition (15.1%, e.g., *fyuA* and *irp2*) (Table S3). Several VGs related to the invasion of host barriers (*ibeBC*), evasion of the immune system (i.e., resistance to phagocytosis (*traT*) and serum (*iss*)), production of toxins (hemolysins) and autotransporters (*upaG* and *ehaB*), resistance to tellurium (*terC*) and acidic conditions (*gad*) were also encoded in the genome (Table S3). *In silico* plasmid typing identified two F-type (FIB and FII) and one Col-like replicons.

### 3.2. Characterization and Comparative Analysis of the pEc355340_NDM-5 Plasmid

**Figure 1:**
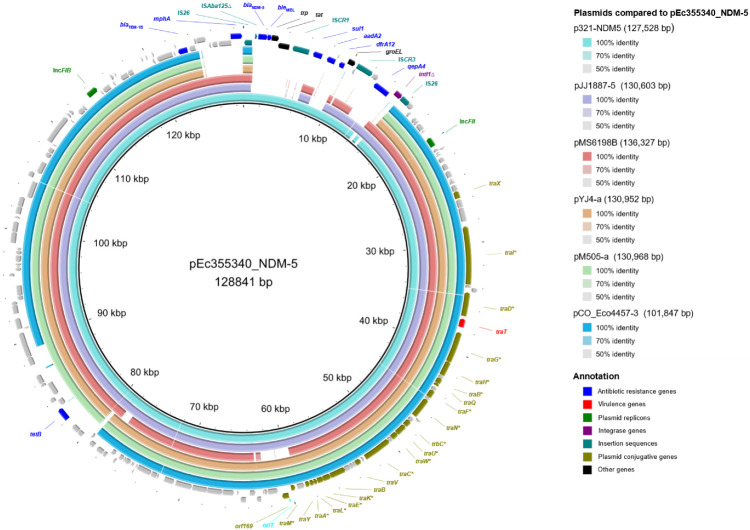
Plasmid sequence comparisons of pEc355340_NDM-5 with similar F-type plasmids identified by a discontinuous megaBLAST using BRIG software. The reference sequence was pEc355340_NDM-5, while rings, from inward to outward, represent the plasmids p321-NDM5 (CP076302), pJJ1887-5 (CP014320), pMS6198B (CP015836), pYJ4-a (AP023232), pM505-a (AP023221) and pCO_Eco4457-3 (CP049970). Gradient colour of the rings represents sequence similarity to the reference plasmid. The arrows in the two most outward rings represent all the coding sequences identified by RAST (https://rast.nmpdr.org/rast.cgi) in pEc355340_NDM-5. Annotation for several coding sequences is available in the figure and are colour-coded according with their function. The genes considered essential for conjugative transfer by Fernandez-Lopez et al. [20], are indicated with an asterisk (*). *oriT* was identified by oriTfinder (https://bioinfo-mml.sjtu.edu.cn/oriTfinder/) and is highlighted in light blue.

*bla*_NDM-5_ was located on a 128 kb multi-replicon FIB-FII plasmid (herein designated pEc355340_NDM-5) that carried eight of the nine ARGs identified in the Ec355340 genome and the virulence gene *traT*. Based on RAST annotation, genes involved in carbohydrate metabolism (e.g., *scrB* and *fruK*), RNA metabolism, plasmid addiction systems (*pemI*, *pemK* and *vapC*) and commensurate regulon activation (*rob*) were also identified in pEc355340_NDM-5. The genes necessary for conjugal transfer were detected in the plasmid backbone (Figure 1).

A discontinuous megaBLAST of the pEc355340_NDM-5 sequence showed it was nearly identical to four plasmids previously reported (100% coverage and approximately 99% identity) (Table S4). Among these, the plasmids pHN15978-1 (MK291500), FDAARGOS_448 (CP023959) and AR_452 (CP030329) carried *bla*_NDM-5_ in the same context as pEc355340_NDM-5 and were isolated from *E. coli* in Pakistan (ST156, retail meat), Canada (ST405, urine) and an unknown source (ST156), respectively (Table S4). Plasmid p321-NDM5, isolated in Germany, was very similar to the previous, but with a truncation of the *qepA4* and *intI1*∆ genes (Figure 1). Five other plasmids exhibited a similar backbone to that of pEc355340_NDM-5 (sequence coverage above 70% and identity above 99%) (Table S4), but in these, the region containing the resistance genes was either absent or contained other genes such as *bla*_CTX-M-15_ or *bla*_CTX-M-15_ plus *qnrS1*.

**Figure 2:**
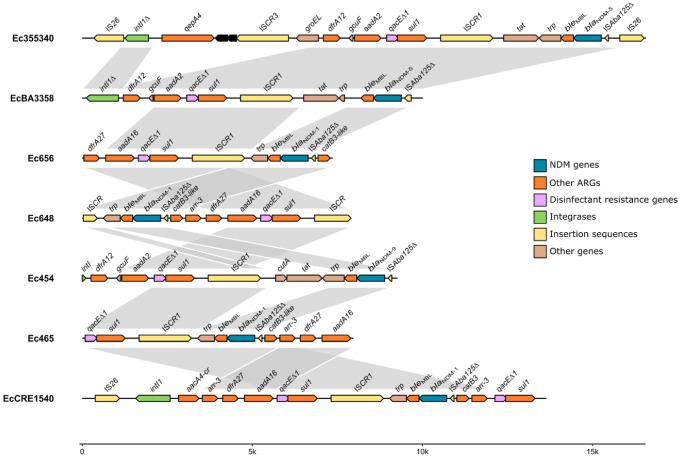
Schematic representation of the *bla*_NDM_-containing complex class 1 integron found in the clinical strain characterized in this study (Ec355340) and representative complex integrons identified in *E. coli* genomes affiliated to ST156 from PATRIC database (https://www.patricbrc.org/ accessed on 1 January 2022). This image was built using the R package gggenomes (https://github.com/thackl/gggenomes accessed on 1 January 2022).

In pEc355340_NDM-5 and similar plasmids, the analysis of the *bla*_NDM-5_ genetic context showed its association with a complex class 1 integron of approximately 14 kb in size (Figure 2). The genes *qepA4*, *dfrA12*, *aadA2* and *sul1* were co-located in this integron. *bla*_NDM-5_ was downstream of an IS*CR*-element with genes encoding a cytochrome c-type biogenesis protein (*dsbD*) and a phosphoribosylanthranilate isomerase (*trp*) and was flanked by a *ble*_MBL_ and a truncated IS*Aba125* gene (Figure 2). The whole complex class 1 integron was flanked by two IS*26* genes (Figure 2). Interestingly, a BLAST search revealed 48 other plasmids with similar complex integrons (coverage > 70% and identity approximately 99%), and some found in other taxa (i.e., *K. pneumoniae* and *Citrobacter freundii*), though the *qepA4*-IS*CR3*-groEL region was absent (Figure 1, Table S4). Four NDM variants (*bla*_NDM-1_, *bla*_NDM-4_, *bla*_NDM-5_ and *bla*_NDM-16_) were found in these complex class 1 integrons (Table S4).

Mating assays were performed to evaluate the transferability of pEc355340_NDM-5. Despite several attempts, no transconjugants were identified. On the other hand, the transformation of pEc355340_NDM-5 into *E. coli* TOP10 resulted in a phenotype of resistance to all beta-lactams tested and sulfamethoxazole-trimethoprim in the host strain (Table 1, TOP10::pEc355340_NDM-5). In addition, the characterization of an Ec355340-derived strain, from which the plasmid was curated (herein designated Ec355340∆pNDM-5), revealed susceptibility to third generation cephalosporins, carbapenems and sulfamethoxazole-trimethoprim (Table 1).

### 3.3. Clonal Background Analysis

To better understand the role of ST156 in NDM dispersion, we analysed the 138 available *E. coli* genomes belonging to this clonal lineage (Table S5), which were from 25 different countries, particularly China (43.5%) and the USA (12.3%) (Figure S1). Most strains were isolated from animal reservoirs (52.2%) and to a lesser extent from humans (37.0%, either clinical (20.3%) or commensal strains (5.8%)) and environmental settings (7.2%) (Table S5). *bla*_NDM_ genes were present in 41 of the genomes (30%), particularly the variants *bla*_NDM-1_ (17 genomes), *bla*_NDM-5_ (11 genomes) and *bla*_NDM-9_ (13 genomes). Most were isolated from animals in animal production facilities in China (n = 29 genomes, Table S5). *mcr-1* was co-carried in 23 of these genomes. The analysis of the genetic environment of *bla*_NDM_ genes showed their association to complex class 1 integrons in at least 20 of the genomes (48.8%), irrespective of the NDM variant identified (Table S5).

The SNPs analysis of all ST156 genomes showed that genomes carrying *bla*_NDM_ were scattered along the SNP tree with no clear pattern regarding their isolation source or geographical location (Figure S2). Ec355340 clustered with the *bla*_NDM-5_-positive *E. coli* AR_452 (collected from a human source), exhibiting identical ARGs, VGs and plasmids profiles (Figure S2, Table S5). The whole-genome comparison of Ec355340 with the two closest genomes according to the SNP analysis, EcAR_452 and Ec89PenNDM (isolated from a penguin in Brazil), yielded ANI values of 99.98% and 99.80%, respectively (Table S6).

The genomes’ antibiotic resistance arsenal varied greatly across this ST as well as the VGs and plasmid content (Figure S2). However, the dendrogram built using the combined presence/absence data of ARGs, VGs and plasmids showed a clear separation in two large clusters (Figure S3). Most NDM-positive ST156 strains (thirty-nine out of forty-two) were present in cluster II, except for three strains: Ec355340, EcAR_452 and BA3358, the last being isolated from a bloodstream infection in India (Figure S3). Higher median values of total number per genome of VGs (14.0 vs. 7.0) and ARGs (17.0 vs. 6.5) were observed for cluster II comparative to cluster I. The gene *mcr-1* was only identified in cluster II and *bla*_CTX-M_ genes were far more prevalent in this cluster (67.8% were *bla*_CTX-M_-positive genomes) than in cluster I (22.0%) (Figure S3). Genes conferring resistance to aminoglycosides and trimethoprim were also more prevalent in cluster II (96.6% vs. 56.0% in cluster I and 87.4% vs. 52.0%, respectively). The genes *ipfA* and *terC* were present in all *bla*_NDM_-containing ST156 genomes, where *iss*, *gad*, *hra* and *astA* were identified in >70%, while *hlyF*, *cvaC* and *estC* were only identified in cluster II and *iucC* and *iutA* were almost exclusive to this group (Figure S3).

To understand *bla*_NDM-5_ dissemination across different *E. coli* lineages and the importance of ST156 in the spread of this variant, we retrieved *E. coli* genomes carrying *bla*_NDM-5_ from the PATRIC database (n = 431; Table S7). These were obtained from 24 different countries from all continents, though most genomes were reported from China (73.1%; Figure S1). Most genomes were from strains isolated from animals (43.6%) and humans (42.7%, from which 39.7% were of a clinical origin), while a small subset was retrieved from environmental samples (7.9%) (Table S7). The MLST analysis of this dataset indicated the existence of at least 100 distinct STs associated to NDM-5, of which ST167 (84 genomes) was the most prevalent, followed by ST410 (36 genomes) and ST10 (27 genomes) [Table S7]. Sequence types of the ST10 clonal complex comprised 35% of the genomes of this dataset. Only 2.8% of the *bla*_NDM-5_-positive *E. coli* genomes belonged to ST156 (Table S7).

## 4. Discussion

The monitoring of antibiotic resistance mechanisms in nosocomial settings is relevant for understanding their dissemination and enables the implementation of adequate antibiotic regimens and outbreak control measures. This is particularly important regarding resistance to last-line antibiotics, such as carbapenems, for which few therapeutic options remain available. In this study, we reported for the first time the presence of a *bla*_NDM-5_-carrying strain in Portugal, characterized its genotype and phenotype and conducted comparative genomic analyses to better understand the implications of the genetic context and clonal background in *bla*_NDM-5_ dissemination.

The identification of *bla*_NDM-5_ in Portugal, as it represents a shift in evolutionary terms towards higher levels of carbapenem resistance in *bla*_NDM_ carriers, may herald a change in the limited dissemination of NDM enzymes in this country (previously only NDM-1 had been reported) [12,13,14,15,16].

A plethora of VGs were identified in Ec355340, some of which were particularly relevant for extraintestinal pathogenesis. In fact, despite having been isolated in the context of a urinary tract infection, Ec355340 presented VGs that may enable it to provoke other serious extraintestinal infections, i.e., systemic bloodstream infection and meningitis (e.g., genes involved in a host’s immune evasion and invasion of the blood–brain barrier) [21,22].

The probable origin of Ec355340 and the transmission route to the patient from whom it was isolated was difficult to pinpoint. Data from Enterobase indicated that ST156 isolates (n = 498 strains) were retrieved from animal (32.5%) and human hosts (24.7%), while a small subset were retrieved from environmental settings (4.6%; Enterobase, https://enterobase.warwick.ac.uk/; accessed on February 2022). In Portugal there are few reports on this ST, mostly from animal origin (e.g., JACBWE000000000, [23]), though it was also recovered from wastewater [24] and clinical human samples [25].

*bla*_NDM-5_-carrying *E. coli* genomes were found to be heterogeneously dispersed through multiple STs, which was consistent with previous epidemiological reports that did not provide evidence of the preferential association of *bla*_NDM_ to specific high-risk clones [8]. In fact, ST156’s low prevalence among the *bla*_NDM-5_ positive *E. coli* genomes (together with the absence of SNP sub-clusters of *bla*_NDM-5_ genomes in ST156) implied that this specific ST was not a major player in *bla*_NDM-5_ dissemination, although in general the ST10 clonal group seemed to be involved. Nonetheless, the ST156 strains included in this analysis had a heterogeneous ARG arsenal (most with a predicted MDR phenotype), with some co-harbouring ARGs conferring resistance to last-resort antibiotics, i.e., carbapenems and polymyxins. In fact, a recent metanalysis confirmed ST156 as one of the strains most commonly associated with co-resistance to carbapenems and polymyxins [26]. Among the plasmid-encoded colistin resistance mechanisms identified, *mcr-1* was the most identified [26], which was in line with the high co-carriage of *mcr-1* and *bla*_NDM_ in *bla*_NDM_-positive ST156 genomes (56%) in our analysis. Since colistin has been recently re-introduced into clinical practice, its use can lead to the selection of bacterial lineages that co-carry *bla*_NDM_ and *mcr-1*. Therefore, an increasing prevalence of clones such as *E. coli* ST156 might be expected in the future, along with a higher contribution of these clones for *bla*_NDM_ dissemination.

The hierarchical clustering of genotype data from ST156 genomes showed Ec355340 and most of the other NDM-positive genomes in distinct clusters, with the second cluster displaying richer ARGs and VGs profiles. This could be related to (i) the association of *bla*_CTX-M_ and *bla*_NDM_ genes to multiresistance plasmids [6,27], (ii) regional patterns of resistance and virulence, since 67.8% of the genomes from this cluster were isolated from China, and (iii) the presence of integrons in these strains, since cassettes conferring resistance to aminoglycosides and trimethoprim (abundant in this cluster) are frequently identified in integrons.

*bla*_NDM-5_ was identified on a multi-replicon FIB-FII plasmid in Ec355340. Plasmids are regarded as the main players in *bla*_NDM_ dissemination worldwide, particularly IncX3 plasmids, though multi-replicon F-type and IncC plasmids are also commonly implicated [8]. The co-existence of more than one replicon in a plasmid is proposed to favour its dissemination by increasing its host range [28], and it can also allow plasmids with incompatible replicons to be acquired by the host cell, provided that that replication is being controlled by a compatible replicon [29]. In fact, when associated to FIA or FIB, FII replicons (when not responsible for replication control) can diverge and give rise to replicon variants compatible with other F-family replicons [29]. Such a strategy facilitates the acquisition of multiple plasmids within the same cell and may facilitate *bla*_NDM_ dissemination by co-selection mechanisms. Despite the promiscuity of IncF plasmids, the conjugal transfer of pEc355340_NDM-5 was not observed under the conditions tested. Nonetheless, the analysis of the plasmid backbone identified the genes necessary for conjugal transfer [20]. In fact, Baloch and colleagues observed a transfer of the plasmid pHN15978 (100% identical to pEc355340_NDM-5) to the recipient *E. coli* C600 [30]. Preliminary experiments using different incubation temperatures, donor:recipient ratios and even different recipient strains also yielded negative results (data not shown). Possibly, the distinct experimental conditions applied here (e.g., cell concentration or recipient strain) might not have provided the necessary conditions for the transfer to occur.

*bla*_NDM-5_ was described here in a complex class 1 integron structure. *bla*_NDM_ has been described in several genetic contexts, though it is usually flanked by IS*Aba125* and *ble*_MBL_ and inserted in transposons such as Tn*125* and other composite transposons [8]. Interestingly, the association of *bla*_NDM_ to complex class 1 integrons has only been clearly stated in a few studies [31,32,33]. In a study analysing publicly available genomes, Acman and colleagues frequently identified IS*CR1* downstream of *bla*_NDM_, but the presence of integrase genes in a 50-kb radius of the *bla*_NDM_ location was only confirmed in 15 contigs of the 6155 genomes analysed [34]. In opposition, Wang and colleagues, in a large characterization effort of bacteria isolated from poultry-associated samples, identified *bla*_NDM_ in integron or integron-like genetic environments in 44 of 161 carbapenem-resistant *E. coli* strains [35], suggesting that this association may be more common than expected, or at least in specific compartments. The limited capacity to assemble large multiresistance and modular regions, such as complex integrons, may explain why, in some studies using short-read sequencing, this association cannot be ascertained.

Interestingly, some studies have also observed a tendency of ARGs in *bla*_NDM_ plasmids to be mostly clustered within a complex class 1 integron [32,33]. This highlights the importance of integrons for the acquisition of multiresistance genotypes by plasmids. However, integrons are also tightly regulated and represent a lower fitness cost for the bacterial host than chromosomal (five- to fifteen-fold) or plasmid-encoded resistance alone (two- to seven-fold) [36]. Therefore, more than being simple resistance assembly platforms, the presence of complex integrons in NDM plasmids may reduce their fitness cost and favour their successful dissemination. The low fitness cost associated to the acquisition of *bla*_NDM_ plasmids has been demonstrated, in some cases even leading to an increased growth rate of the bacterial host [37,38,39]. This has clear implications on NDM dissemination since these plasmids not only confer resistance to carbapenems but also favour bacterial proliferation in the absence of antibiotic exposure. In pEc355340_NDM-5, the presence of genes associated to sucrose utilization and biosynthesis of tryptophan may have similar effects on the attenuation of the plasmid cost.

Unlike *bla*_KPC_, whose worldwide distribution is known to be driven by its association with the highly successful *Klebsiella pneumoniae* ST258 clone [40], the major drivers of *bla*_NDM_ dissemination are not well elucidated. Previous analysis has shown that the association to promiscuous plasmids appears to be much more relevant than the acquisition of NDM genes by any successful clonal lineage [8]. The analysis employed in this study supports this hypothesis (particularly to *bla*_NDM-5_) but also highlights other possible important players, namely complex class 1 integrons. Future studies should, therefore, address the actual fitness burden imposed by *bla*_NDM_ in different genetic contexts to understand potential fitness cost mitigation strategies related to mobile genetic elements such as integrons. The use of long-read sequencing technologies or the complete sequencing of plasmid sequences should also facilitate the full characterization of the *bla*_NDM_ context, further unravelling the possible association to complex class 1 integrons. Studies evaluating NDM dissemination in Portugal are also needed to monitor the prevalence of these carbapenemases in Portuguese settings.

## Figures and Tables

**Table 1:** Minimum inhibitory concentrations determined by VITEK^®^ 2 for the clinical strain identified in this study (Ec355340::pNDM-5), the plasmid-cured strain (Ec355340∆pNDM-5), the transformant (TOP10::pNDM-5) and the recipient electrocompetent cells (TOP10).

Antibiotic Class	Antibiotics		Ec355340∆pNDM-5	Ec355340::pNDM-5		TOP10	TOP10::pNDM-5			
3rd generation penicillins	Ampicillin		≥32	≥32		8	≥32			
	Amoxicillin/clavulanic acid		≥32	≥32		4	≥32			
4th generation penicillins	Piperacillin/tazobactam		≤4	≥128		≤4	≥128			
2nd generation cephalosporins	Cefuroxime		16	≥64		8	≥64			
	Cefuroxime axetil		16	≥64		8	≥64			
3rd generation cephalosporins	Cefotaxime		≤1	≥64		≤1	≥64			
	Ceftazidime		≤1	≥64		≤1	≥64			**Resistant**
4th generation cephalosporins	Cefepime		≤1	8		≤1	16			**Intermediate**
Carbapenems	Ertapenem		≤0.12	≥8		≤0.12	≥8			**Susceptible**
	Meropenem		≤0.25	≥16		≤0.25	≥16			
Aminoglycosides	Amikacin		≤2	≤2		≤2	≤2			
	Gentamicin		≤1	≤1		≤1	≤1			
Fluoroquinolones	Ciprofloxacin		≥4	≥4		≤0.25	≤0.25			
-	Fosfomycin		≤16	≤16		≤16	≤16			
-	Nitrofurantoin		32	≤16		≤16	≤16			
Polymyxins	Colistin		≤0.5	≤0.5		≤0.5	≤0.5			
Sulphonamides	Trimethoprim/sulphamethoxazole		≤20	≥320		≤20	≥320			

## Data Availability

The draft-genome sequences (Ec355340 and Ec355340∆pNDM-5) and the pEc355340_NDM-5 complete sequence are available in GenBank under the BioProject PRJNA856461 and accession OP046713, respectively.

## Supplementary Materials

The following supporting information can be downloaded at: https://www.mdpi.com/article/10.3390/microorganisms10112243/s1, Table S1: Primers and thermocycling conditions used in this study; Table S2: Quality metrics of the sequenced genomes obtained in this study; Table S3: Virulence genes detected in strain Ec355340; Table S4. TOP100 most similar sequences to the plasmid pEc355340_NDM-5; Table S5: *E. coli* genomes of the sequence type 156 retrieved from PATRIC database; Figure S1. Geographical distribution of *E. coli* ST156 genomes and NDM-5 positive *E. coli* genomes deposited in PATRIC database and analysed in this study; Figure S2: Core SNP tree built from the *E. coli* ST156 genomes available in PATRIC; Table S6: Average nucleotide identity matrix for the *E. coli* ST156 genomes closest to Ec355340; Figure S3: Hierarchical clustering of *E. coli* ST156 genomes based on the presence/absence of ARGs, VGs and plasmid; Table S7: NDM-5-positive *E. coli* genomes available in PATRIC and corresponding metadata. References [41,42,43,44,45] were cited in the Supplementary Materials.
