# Supplementary material for: Report and Comparative Genomics of an NDM-5-Producing Escherichia coli in a Portuguese Hospital: Complex Class 1 Integrons as Important Players in blaNDM Spread
Source: Microorganisms. 2022 Nov 12;10(11):2243. doi: 10.3390/microorganisms10112243 (PMC9693474; doi:10.3390/microorganisms10112243)
Supplement: Supplementary file 1 [file microorganisms-10-02243-s001.zip › Supplementary material.pdf]

Supplementary material

Report and comparative genomics of NDM-5-producing *Escherichia coli* in a Portuguese hospital: complex class 1 integrons as important players in *bla*$_{NDM}$ spread

Rafael D. S. Tavares [1,2], Marta Tacão [2,*], Elmano Ramalheira [3], Sónia Ferreira [3,4,5], Isabel Henriques [1]

[1] University of Coimbra, Centre for Functional Ecology (CFE), Department of Life Sciences, Calçada Martim de Freitas, 3000-456 Coimbra, Portugal

[2] Centre for Environmental and Marine Studies (CESAM) and Department of Biology, University of Aveiro, Campus Universitário Santiago, 3810-193 Aveiro, Portugal

[3] Serviço de Patologia Clínica, Centro Hospitalar do Baixo Vouga-EPE, Avenida Artur Ravara, 3810-501 Aveiro, Portugal

[4] Department of Medical Sciences, University of Aveiro, Campus Universitário Santiago, 3810-193 Aveiro, Portugal

[5] Instituto de Educação e Cidadania, Largo da Igreja, 3770-033 Mamarrosa, Aveiro, Portugal

* Correspondence: martat@ua.pt;

Table S1. Primers and thermocycling conditions used in this study.

Primers	Target	Primer sequence (5'- 3')	Thermocycling program[1]	Reference	Positive control
NDM_fwd<br>NDM_rev	bla_{NDM}	GGT TTG GCG ATC TGG TTT TC<br>CGG AAT GGC TCA TCA CGA TC	10 min at 94 ºC; (30 s at 94 ºC, 40 s at 52 ºC, 50 s at 72 ºC) for 36 cycles; 7 min at 72 ªC	[41]	*Enterobacter* CR8[2]
BOXA1R	BOX elements	CTA CGG CAA GGC GAC GCT GAC G	7 min at 94 ºC; (1 min at 94 ºC, 1 min at 53 ºC, 8 min at 65 ºC) for 30 cycles; 16 min at 65 ºC	[42]	-
TEM_fwd<br>TEM_rev	bla_{TEM}	CAT TTC CGT GTC GCC CTT ATT C<br>CGT TCA TCC ATA GTT GCC TGA C	5 min at 94 ºC; (40 s at 94 ºC, 40 s at 60 ºC, 1 min at 72 ºC) for 30 cycles; 7 min at 72 ºC	[43]	*E. coli* Ec355340[3]
tetB_fwd<br>tetB_rev	*tetB*	TCA TTG CCG ATA CCA CCT CAG<br>CCA ACC ATC ATG CTA TTC CAT CC	5 min at 94 ºC; (30 s at 94 ºC, 30 s at 53 ºC, 30 s at 72 ºC) for 30 cycles; 7 min at 72 ºC	[44]	*E. coli* Ec355340[3]
intI1_fwd<br>intI1_rev	*intI1*	CCT CCC GCA CGA TGA TC<br>TCC ACG CAT CGT CAG GC	5 min at 94 ºC; (30 s at 94 ºC, 30 s at 55 ºC, 30 s at 72 ºC) for 30 cycles; 7 min at 72 ºC	[45]	*E. coli* Ec355340[3]
R1_fwd[4]<br>R1_rev[4]	pEc355340_NDM-5	GCA CTG TTG CAA ATA GTC GGT<br>GTC GGT AAC CTC GCG CAT A	5 min at 94 ºC; (30 s at 94 ºC, 30 s at 60 ºC, 90 s at 72 ºC) for 30 cycles; 7 min at 72 ºC	This study	-
R2_fwd[4]<br>R2_rev[4]	pEc355340_NDM-5	TTT CGC GTC AGG GAT GGA AG<br>TTT TCT GAA CCA GGT CGC CA	5 min at 94 ºC; (30 s at 94 ºC, 30 s at 60 ºC, 90 s at 72 ºC) for 30 cycles; 7 min at 72 ºC	This study	-
R3_fwd[4]<br>R3_rev[4]	pEc355340_NDM-5	GGA AAA AGA GCG CGC TGA AA<br>CAC ATA CCA GAA GCC GTC GT	5 min at 94 ºC; (30 s at 94 ºC, 30 s at 60 ºC, 90 s at 72 ºC) for 30 cycles; 7 min at 72 ºC	This study	-

[1]PCR reactions with a 25 µL-volume were prepared in autoclaved milli-Q water with a concentration of 0.05 U/µL of NZYTaq 2x Green Master Mix (NzyTech, Portugal) and 0.3 µM of each primer. The only exception was in BOX-PCR reaction were the concentration of primer BOXA1R was 0.4 µM.

[2]Bacterial isolate characterized by Teixeira *et al*. [14].

[3]Bacterial isolate characterized in this study. Used in this PCR assays as control since whole-genome sequencing data confirmed the presence of these genes and a comparative strategy to the original strain was used.

[4]Primers used for the assembly of pEc355340_NDM-5.

Table S2. Quality metrics of the sequenced genomes obtained in this study as determined by RAST tool (https://rast.nmpdr.org/rast.cgi).

**Quality metrics**	**Ec355340**	**Ec355340ΔpNDM-5**
Putative genome size (bp)	5,004,892	4,884,878
Number of contigs (>500 bp)	64	57
GC content (%)	50.9	50.9
Shortest contig size (bp)	597	597
Median sequence size (bp)	31,036	34,400
Mean sequence size (bp)	78,201.4	85,699.6
Longest contig size (bp)	491,699	491,699
N50	215,417	208,412
L50	8	9
Predicted coding sequences	4,867	4,724

Table S3. Virulence genes detected in strain Ec355340 by VirulenceFinder and/or VFanalyzer. The number of genes identified per category is indicated within square brackets.

VF class	Virulence factors	Genes identified
**Adherence [29]**	CFA/I fimbriae [3]	*cfaA*
		cfaB
		cfaC
	E. coli common pilus (ECP) [6]	*ecpA*
		ecpB
		ecpC
		ecpD
		ecpE
		ecpR
	E.coli laminin-binding fimbriae (ELF) [4]	*elfA*
		elfC
		elfD
		elfG
	EaeH [1]	*eaeH*
	Hemorrhagic *E.coli* pilus (HCP) [3]	*hcpA*
		hcpB
		hcpC
	P fimbriae [3]	*papC*
		papD
		papH
	Type I fimbriae [8]	*fimA*
		fimC
		fimD
		fimE
		fimF
		fimG
		fimH
		fimI
	Long polar fimbriae [1]	*ipfA*
**Autotransporter [2]**	EhaB [1]	*ehaB*
	UpaG adhesin [1]	*upaG/ehaG*
**Invasion [2]**	Invasion of brain endothelial cells (Ibes) [2]	*ibeB*
		ibeC
**Iron uptake [11]**	Yersiniabactin siderophore [11]	*fyuA*
		irp1
		irp2
		ybtA
		ybtE
		ybtP

		ybtQ
		ybtS
		ybtT
		ybtU
		ybtX
**Non-LEE encoded TTSS effectors [6]**	EspL1 [1]	*espL1*
	EspL4 [1]	*espL4*
	EspR1 [1]	*espR1*
	EspX1 [1]	*espX1*
	EspX4 [1]	*espX4*
	EspX5 [1]	*espX5*
**Secretion system [17]**	ACE T6SS [17]	-
		aec15
		aec16
		aec17
		aec18
		aec19
		aec22
		aec23
		aec24
		aec25
		aec26
		aec27/clpV
		aec28
		aec29
		aec30
		aec31
		aec32
**Toxins [1]**	Hemolysin/cytolysin A [1]	*hlyE/clyA*
**Serum resistance [2]**	LPS rfb locus (*Klebsiella*) [1]	-
	Increased serum survival (Iss) protein [1]	*iss*
**Immune evasion [1]**	Outer membrane protein complement resistance [1]	*traT*
**Others [2]**	Glutamate decarboxylase (acid resitance) [1]	*gad*
	Tellurium ion resistance protein [1]	*terC*

Table S4. TOP100 most similar sequences to the plasmid pEc355340_NDM-5. The overall comparison was performed by a discontinuous megaBLAST using the pEc355340_NDM-5 full sequence as query. The existence of similar complex class 1 integrons associated with bla_{NDM} was assessed in this dataset by megaBLAST using the sequence of the complex integron identified in pEc355340_NDM-5 (14 kb). Integron arrays were manually annotated, and plasmid replicon types of relevant sequences were identified using PlasmidFinder 2.1. Metadata was also extracted from GenBank.

Table S5. E. coli genomes of the sequence type 156 retrieved from PATRIC database (https://www.patricbrc.org/). Metadata was obtained from PATRIC, the original GenBank records or from the reporting publication. Identification of antibiotic resistance genes, virulence genes, plasmid replicons and serotype were performed by tools at the Center for Genomic Epidemiology (https://cge.cbs.dtu.dk/services/). PathogenFinder was used to predict the potential pathogenicity of each genome. The blaNDM genetic context was determined by manual inspection of the contig where the gene was identified in PATRIC using the tool “Genome Browser”. Class 1 integrons were screened by BLAST searches of intI1 gene (accession NC_022375.1) against our genome dataset (https://www.patricbrc.org/app/BLAST). Variable regions were identified, when possible, by manual inspection of the intI1-carrying contigs.

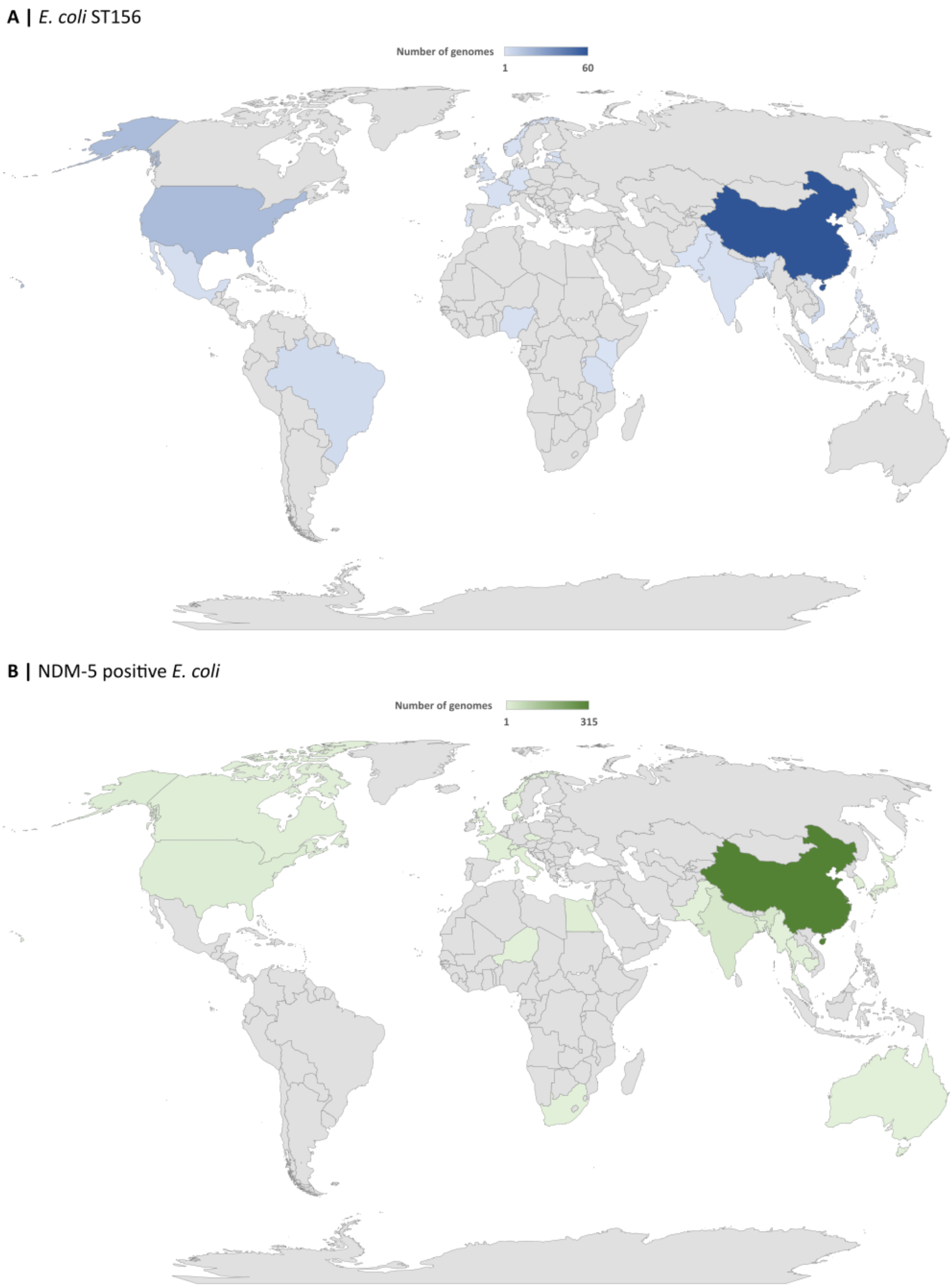

Figure S1. Geographical distribution of (A) E. coli ST156 genomes (n= 138) and (B) NDM-5 positive E. coli genomes (n=431) deposited in PATRIC database (https://www.patricbrc.org/) and analysed in this study. For 9 and 28 genomes of each respective dataset, information regarding country of isolation was not available.

Figure S2. Core SNP tree built from the E. coli ST156 genomes available in PATRIC, using REALPHY 1.13 with default settings (https://realphy.unibas.ch/realphy/). The genome of E. coli AR_452 was used as reference. Metadata related to NDM presence, isolation and geographical source of each genome is represented. The number of ARGs per genome and class of antibiotics to which it confers resistance is identified in a stack barplot while the number of VGs and plasmid replicon detected are displayed in simple barplots. The blue square indicates the cluster that includes Ec355340 and its closest genomes. This figure was built using iTOL version 6 (https://itol.embl.de/).

Table S6. Average nucleotide identity (ANI) matrix for the *E. coli* ST156 genomes closest to Ec355340. Values are presented in percentage and in brackets are indicated the percentage of aligned nucleotides. Pairwise ANI calculations were performed at JSpeciesWS (Ribocon, https://jspecies.ribohost.com/jspeciesws/)

	**Ec355340**	**Ec AR_452**	**Ec 89PenNDM**	**Ec 112**
**Ec355340**	-	99.98 (99.03)	99.80 (92.72)	99.76 (91.63)
**Ec AR_452**	-	-	99.81 (94.13)	99.76 (93.44)
**Ec 89PenNDM**	-	-	-	99.63 (90.87)
**Ec 112**	-	-	-	-

Figure S3. Hierarchical clustering of *E. coli* ST156 genomes based on the presence/absence of ARGs, VGs and plasmid. The resemblance matrix was built using the Jaccard coefficient and the dendrogram was built using UPMGA as the clustering method in Primer v.6. The heatmap, plotted using R's ggplot2, represents presence/absence of all the genes identified in at least one of these genomes. NDM variants identified in each genome are indicated by colored squares behind each label. The 2 main clusters identified from this analysis are indicated as cluster I and cluster II.

Table S7. NDM-5 positive E. coli genomes available in PATRIC and corresponding metadata. Genomes were selected based on “PATRIC Local Family” feature. Specificity to NDM-5 variant was ensured by BLAST analysis (only perfect hits were considered). The sequence type affiliation for each genome was obtained using the MLST 2.0 tool (https://cge.cbs.dtu.dk/services/MLST/).
