# Supplementary figures and images for: Report and Comparative Genomics of an NDM-5-Producing Escherichia coli in a Portuguese Hospital: Complex Class 1 Integrons as Important Players in blaNDM Spread
Source: Microorganisms. 2022 Nov 12;10(11):2243. doi: 10.3390/microorganisms10112243 (PMC9693474; doi:10.3390/microorganisms10112243)

Tree scale: 0.001

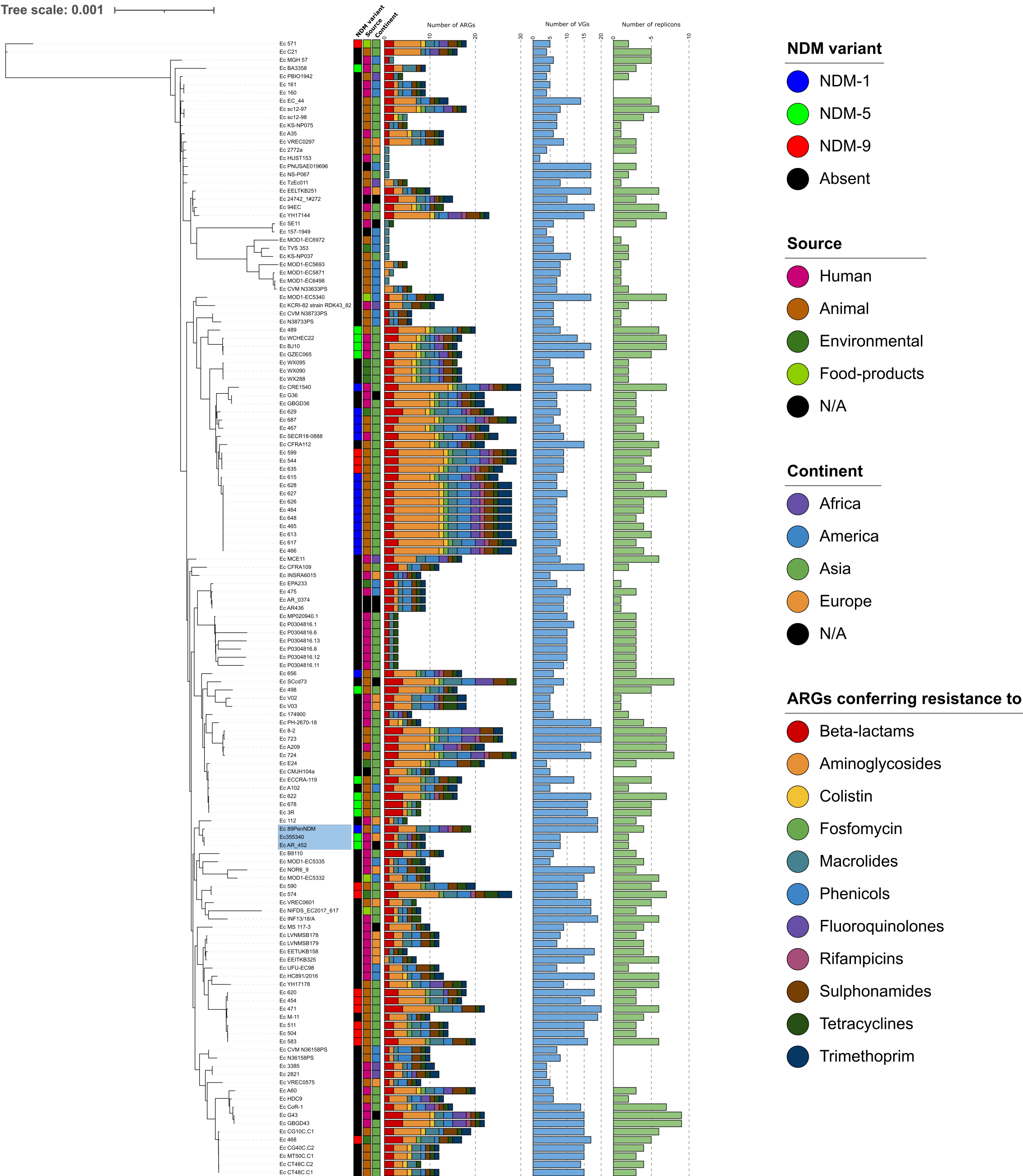

Supplement: Supplementary file 1 [file microorganisms-10-02243-s001.zip › Figure S2.pdf]

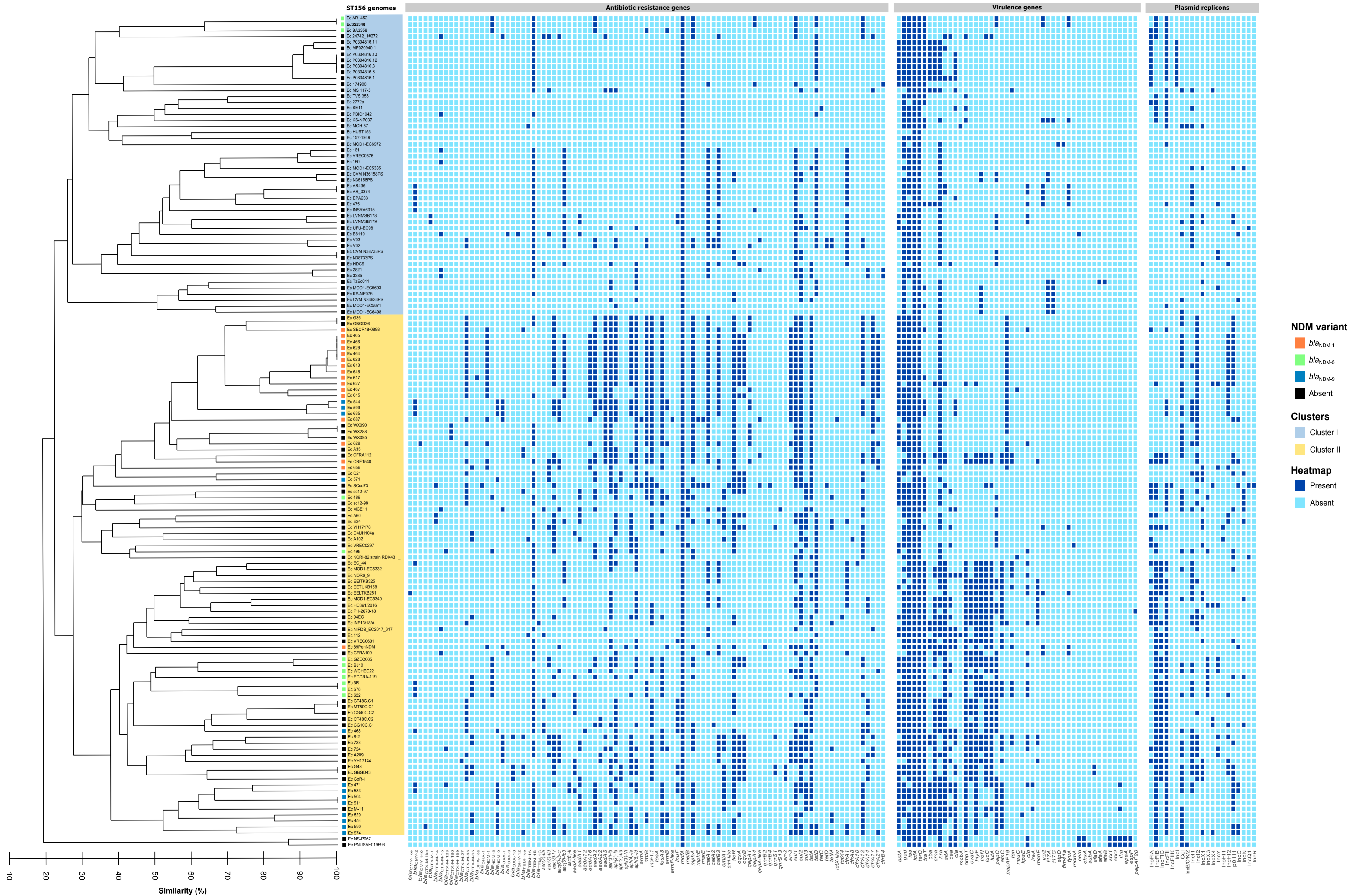

Supplement: Supplementary file 1 [file microorganisms-10-02243-s001.zip › Figure S3.pdf]
